# Tetra­kis(5,7-dimethyl­quinolin-8-olato-κ^2^ *N*,*O*)hafnium(IV) dimethyl­formamide disolvate

**DOI:** 10.1107/S1600536811038311

**Published:** 2011-09-30

**Authors:** J. Augustinus Viljoen, Hendrik G. Visser, Andreas Roodt

**Affiliations:** a Department of Chemistry, University of the Free State, P.O. Box 339, Bloemfontein, 9300, South Africa

## Abstract

In the title compound, [Hf(C_11_H_10_NO)_4_]·2C_3_H_7_NO, the Hf^IV^ atom is coordinated by four *N*,*O*-donating bidentate 5,7-dimethyl-8-quinolino­late (Ox^−^) ligands arranged to give a distorted square-anti­prismatic coordination polyhedron. The average Hf—O and Hf—N distances are 2.098 and 2.298 Å, respectively, and the average O—Hf—N bite angle is 70.2°. The crystal packing is controlled by π–π inter­actions between Ox^−^ ligands of neighbouring mol­ecules, giving rise to a three-dimensional supra­molecular grid network. The inter­planar distances vary from 3.441 (1) to 3.509 (1) Å, while the centroid–centroid distances vary from 3.688 (2) to 3.759 (12) Å. A non-classical C—H⋯O hydrogen bond is observed between the complex and one of the solvate mol­ecules.

## Related literature

For related literature on Hf^IV^ and Zr^IV^ *N,O*- and *O,O′*-dike­to­nato complexes, see: Viljoen *et al.* (2008 ▶, 2009*a* ▶,*b* ▶, 2010*a* ▶,*b* ▶); Steyn *et al.* (2008 ▶, 2011 ▶). For relevant studies on *N,O*- and *O,O′*-bidentate ligands with other transition metal atoms, see: Graham *et al.* (1991 ▶); Mtshali *et al.* (2006 ▶); Roodt *et al.* (2011 ▶); Schutte *et al.* (2008 ▶); Steyn *et al.* (1997 ▶); Van Aswegen *et al.* (1991 ▶); Van der Westhuizen *et al.* (2010 ▶).
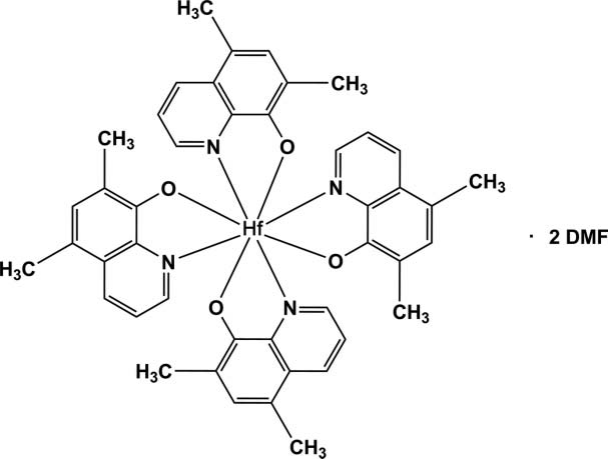


## Experimental

### 

#### Crystal data

[Hf(C_11_H_10_NO)_4_]·2C_3_H_7_NO
*M* *_r_* = 1013.48
Monoclinic, 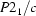

*a* = 9.978 (2) Å
*b* = 16.059 (3) Å
*c* = 28.509 (5) Å
β = 101.582 (1)°
*V* = 4475.2 (15) Å^3^
*Z* = 4
Mo *K*α radiation
μ = 2.39 mm^−1^
*T* = 100 K
0.26 × 0.22 × 0.18 mm

#### Data collection

Bruker X8 APEXII 4K Kappa CCD diffractometer
Absorption correction: multi-scan (*SADABS*; Bruker, 2004 ▶) *T*_min_ = 0.576, *T*_max_ = 0.673
76225 measured reflections
11107 independent reflections
8976 reflections with *I* > 2σ(*I*)
*R*_int_ = 0.058

#### Refinement

*R*[*F* ^2^ > 2σ(*F* ^2^)] = 0.029
*wR*(*F* ^2^) = 0.066
*S* = 1.04
11107 reflections
581 parameters
H-atom parameters constrained
Δρ_max_ = 1.44 e Å^−3^
Δρ_min_ = −0.67 e Å^−3^

### 

Data collection: *APEX2* (Bruker, 2005 ▶); cell refinement: *SAINT-Plus* (Bruker, 2004 ▶); data reduction: *SAINT-Plus*; program(s) used to solve structure: *SIR92* (Altomare *et al.*, 1999 ▶); program(s) used to refine structure: *SHELXL97* (Sheldrick, 2008 ▶); molecular graphics: *DIAMOND* (Brandenburg & Putz, 2005 ▶); software used to prepare material for publication: *WinGX* (Farrugia, 1999 ▶).

## Supplementary Material

Crystal structure: contains datablock(s) I, global. DOI: 10.1107/S1600536811038311/wm2534sup1.cif

Structure factors: contains datablock(s) I. DOI: 10.1107/S1600536811038311/wm2534Isup2.hkl

Additional supplementary materials:  crystallographic information; 3D view; checkCIF report

## Figures and Tables

**Table 1:** Hydrogen-bond geometry (Å, °)

*D*—H⋯*A*	*D*—H	H⋯*A*	*D*⋯*A*	*D*—H⋯*A*
C42—H42⋯O5	0.93	2.55	3.348 (4)	144

**Fig. 1.**
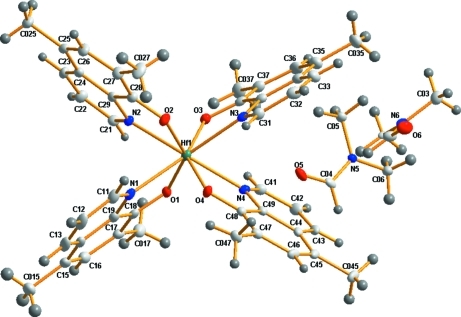
Representation of the molecular title compound with displacement ellipsoids drawn at the 50% probability level. H atoms are presented as small spheres of arbitrary radius.

**Fig. 2.**
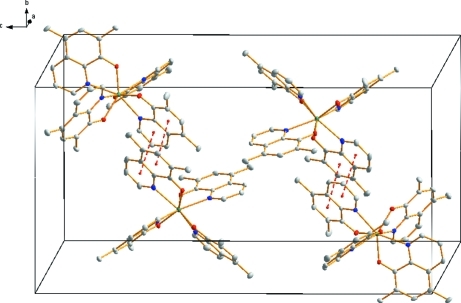
Graphical illustration of π–π interaction and stacking between different Ox--ligands of neighboring molecules to form a three-dimensional network (displacement ellipsoids are drawn at the 50% probability level). Hydrogen atoms and solvent water molecules were omitted for clarity.

## supplementary crystallographic information

### Comment

This study forms part of an ongoing research project that investigates the chelating behaviour of O,O'- and N,O-bidentate ligands with hafnium(IV) and zirconium(IV) for possible separation of these two metals (Steyn et al., (2008, 2011); Viljoen et al., (2008, 2009a, 2009b, 2010a, 2010b). If hafnium and zirconium show differences in their chelating behaviour, either by reaction rates, solubilities, coordination modes, equilibrium behaviour, etc., it could possibly be exploited as a novel separation technique for the two metals. The introduction of N,O-bidentate ligands with the oxine or aminovinylketone backbones significantly influences both steric and electronic properties of transition metals as illustrated by literature examples (Graham et al., 1991; Mtshali et al., 2006; Roodt et al., 2011; Schutte et al., 2008; Steyn et al., 1997; Van Aswegen et al., 1991; Van der Westhuizen et al., 2010).

Red parallelepiped-like crystals of the title compound crystallize with two dimethylformamide solvent molecules in the asymmetric unit (Figure 1). The structure of the title compound is composed of an eight-coordinate Hf(IV) atom in which the four N,O-donating bidentate ligands, 5,7-dimethyl-8-hydroxyquinoline (Ox^-^), are arranged around the metal atom to give a distorted square antiprismatic geometry. The Hf—O and Hf—N bond lengths vary from 2.094 (2) to 2.1036 (19) Å and 2.377 (2) to 2.413 (2) Å, respectivily, and the O—Hf—N bite angles vary from 69.58 (8) to 70.87 (1)°. Only one C—H···O hydrogen bonding interaction is observed between a solvent molecule and one of the oxygen atoms in the complex molecule (Table 1). The molecular units of the title compound are stablilized by π–π interactions between different Ox^-^ ligands of neighbouring molecules, producing a three dimensional supramolecular grid network, with interplaner distances varying between 3.441 (1) and 3.509 (1) Å and centroid-to-centroid distances from 3.668 (2) to 3.759 (2) Å (Figure 2).

### Experimental

Chemicals were purchased from Sigma-Aldrich and used as received. HfCl_4_ (206 mg, 0.64 mmol) was dissolved in a minimal amount of DMF. While stirring this solution at room temperature, another solution of 5,7-dimethyl-8-quinolinol (OxH, C_11_H_11_NO) (445 mg, 2.5 mmol) was dissolved in a minimal amount of DMF and slowly added to the HfCl_4_ solution, resulting in the formation of a bright yellow solution. The solution was left to stand for *ca* a week for reddish crystals to form.

### Refinement

The aromatic, methine, and methyl H atoms were placed in geometrically idealized positions (C—H = 0.93–0.98) and constrained to ride on their parent atoms with *U*_iso_(H) = 1.2*U*_eq_(C) for aromatic and methine, and *U*_iso_(H) = 1.5*U*_eq_(C) for methyl protons. The highest residual electron density was located 1.34 Å from H33 and was essentially meaningless.

### Crystal data

**Table:**

[Hf(C_11_H_10_NO)_4_]·2C_3_H_7_NO	*F*(000) = 2064
*M**_r_* = 1013.48	*D*_x_ = 1.504 Mg m^−^^3^
Monoclinic, *P*2_1_/*c*	Mo *K*α radiation, λ = 0.71073 Å
Hall symbol: -P 2ybc	Cell parameters from 9967 reflections
*a* = 9.978 (2) Å	θ = 3.1–28.1°
*b* = 16.059 (3) Å	µ = 2.39 mm^−^^1^
*c* = 28.509 (5) Å	*T* = 100 K
β = 101.582 (1)°	Parallelepiped, reddish
*V* = 4475.2 (15) Å^3^	0.26 × 0.22 × 0.18 mm
*Z* = 4	

### Data collection

**Table:**

Bruker X8 APEXII 4K Kappa CCD diffractometer	11107 independent reflections
Radiation source: fine-focus sealed tube	8976 reflections with *I* > 2σ(*I*)
graphite	*R*_int_ = 0.058
ω– and φ–scans	θ_max_ = 28.3°, θ_min_ = 4.1°
Absorption correction: multi-scan (*SADABS*; Bruker, 2004)	*h* = −13→13
*T*_min_ = 0.576, *T*_max_ = 0.673	*k* = −21→21
76225 measured reflections	*l* = −38→38

### Refinement

**Table:**

Refinement on *F*^2^	Primary atom site location: structure-invariant direct methods
Least-squares matrix: full	Secondary atom site location: difference Fourier map
*R*[*F*^2^ > 2σ(*F*^2^)] = 0.029	Hydrogen site location: inferred from neighbouring sites
*wR*(*F*^2^) = 0.066	H-atom parameters constrained
*S* = 1.04	*w* = 1/[σ^2^(*F*_o_^2^) + (0.0224*P*)^2^ + 5.254*P*] where *P* = (*F*_o_^2^ + 2*F*_c_^2^)/3
11107 reflections	(Δ/σ)_max_ = 0.004
581 parameters	Δρ_max_ = 1.44 e Å^−^^3^
0 restraints	Δρ_min_ = −0.67 e Å^−^^3^

### Special details

**Table:**

Geometry. All s.u.'s (except the s.u. in the dihedral angle between two l.s. planes) are estimated using the full covariance matrix. The cell s.u.'s are taken into account individually in the estimation of s.u.'s in distances, angles and torsion angles; correlations between s.u.'s in cell parameters are only used when they are defined by crystal symmetry. An approximate (isotropic) treatment of cell s.u.'s is used for estimating s.u.'s involving l.s. planes.
Refinement. Refinement of *F*^2^ against ALL reflections. The weighted *R*-factor *wR* and goodness of fit *S* are based on *F*^2^, conventional *R*-factors *R* are based on *F*, with *F* set to zero for negative *F*^2^. The threshold expression of *F*^2^ > 2σ(*F*^2^) is used only for calculating *R*-factors(gt) *etc*. and is not relevant to the choice of reflections for refinement. *R*-factors based on *F*^2^ are statistically about twice as large as those based on *F*, and *R*- factors based on ALL data will be even larger.

### Fractional atomic coordinates and isotropic or equivalent isotropic displacement parameters (Å^2^)

**Table:**

	*x*	*y*	*z*	*U*_iso_*/*U*_eq_	
C01	0.1539 (4)	0.2498 (2)	0.42031 (16)	0.0448 (10)	
H01	0.2035	0.2827	0.4445	0.054*	
C02	0.3731 (4)	0.1983 (3)	0.40668 (19)	0.0613 (13)	
H02A	0.4073	0.2345	0.4332	0.092*	
H02B	0.407	0.2163	0.3791	0.092*	
H02C	0.403	0.1424	0.4147	0.092*	
C03	0.1559 (5)	0.1477 (3)	0.35772 (15)	0.0507 (11)	
H03A	0.0589	0.1564	0.3529	0.076*	
H03B	0.1764	0.0905	0.3661	0.076*	
H03C	0.1868	0.1607	0.3288	0.076*	
C04	0.7126 (3)	0.2436 (2)	0.44193 (14)	0.0327 (8)	
H04	0.7275	0.2964	0.4306	0.039*	
C05	0.7017 (4)	0.0957 (2)	0.42779 (13)	0.0367 (9)	
H05A	0.6851	0.0961	0.4598	0.055*	
H05B	0.7843	0.0654	0.4272	0.055*	
H05C	0.6263	0.0695	0.4068	0.055*	
C06	0.7343 (4)	0.1912 (2)	0.36369 (13)	0.0416 (9)	
H06A	0.7601	0.2478	0.3592	0.062*	
H06B	0.6502	0.1787	0.3419	0.062*	
H06C	0.8049	0.1544	0.3577	0.062*	
C11	0.3746 (3)	0.42689 (19)	0.76399 (10)	0.0205 (6)	
H11	0.2839	0.4098	0.7595	0.025*	
C12	0.4259 (4)	0.48198 (19)	0.80169 (11)	0.0273 (8)	
H12	0.3685	0.5017	0.8212	0.033*	
C13	0.5599 (4)	0.50671 (19)	0.80984 (11)	0.0267 (8)	
H13	0.5929	0.5437	0.8346	0.032*	
C14	0.6479 (3)	0.47604 (17)	0.78070 (10)	0.0200 (6)	
C15	0.7893 (3)	0.49382 (18)	0.78624 (11)	0.0237 (7)	
C015	0.8620 (4)	0.54853 (19)	0.82691 (11)	0.0310 (8)	
H1A	0.9574	0.5521	0.8258	0.047*	
H1B	0.8522	0.5248	0.857	0.047*	
H1C	0.8225	0.6033	0.8237	0.047*	
C16	0.8590 (3)	0.45965 (19)	0.75367 (11)	0.0246 (7)	
H16	0.9524	0.47	0.7579	0.03*	
C017	0.8763 (3)	0.37667 (19)	0.67860 (11)	0.0220 (7)	
H1D	0.9718	0.3886	0.6895	0.033*	
H1E	0.8443	0.4027	0.6481	0.033*	
H1F	0.8633	0.3175	0.6755	0.033*	
C17	0.7972 (3)	0.40981 (17)	0.71426 (10)	0.0181 (6)	
C18	0.6598 (3)	0.39250 (16)	0.70831 (10)	0.0158 (6)	
C19	0.5860 (3)	0.42315 (17)	0.74224 (10)	0.0173 (6)	
C21	0.6353 (3)	0.19763 (17)	0.73648 (10)	0.0154 (6)	
H21	0.6905	0.2173	0.7162	0.018*	
C22	0.6915 (3)	0.14223 (17)	0.77322 (10)	0.0181 (6)	
H22	0.7817	0.1246	0.7766	0.022*	
C23	0.6120 (3)	0.11425 (17)	0.80407 (10)	0.0177 (6)	
H23	0.6488	0.078	0.8287	0.021*	
C24	0.4751 (3)	0.14027 (16)	0.79851 (10)	0.0146 (6)	
C25	0.3821 (3)	0.11410 (17)	0.82769 (10)	0.0168 (6)	
C025	0.4293 (3)	0.05752 (18)	0.87035 (10)	0.0204 (6)	
H2A	0.3524	0.0426	0.8841	0.031*	
H2B	0.496	0.086	0.8938	0.031*	
H2C	0.4694	0.0081	0.8601	0.031*	
C26	0.2499 (3)	0.14117 (18)	0.81527 (10)	0.0190 (6)	
H26	0.1894	0.1243	0.8343	0.023*	
C027	0.0485 (3)	0.2153 (2)	0.76270 (12)	0.0283 (8)	
H2D	0.0048	0.1831	0.7355	0.042*	
H2E	0.039	0.2735	0.7551	0.042*	
H2F	0.0064	0.2035	0.7894	0.042*	
C27	0.1978 (3)	0.19314 (18)	0.77535 (10)	0.0180 (6)	
C28	0.2880 (3)	0.22049 (17)	0.74737 (10)	0.0148 (6)	
C29	0.4263 (3)	0.19433 (16)	0.75958 (9)	0.0122 (5)	
C31	0.0712 (3)	0.26449 (17)	0.60250 (10)	0.0175 (6)	
H31	0.0411	0.3028	0.6226	0.021*	
C32	−0.0253 (3)	0.22841 (19)	0.56515 (11)	0.0238 (7)	
H32	−0.1177	0.2413	0.5616	0.029*	
C33	0.0175 (3)	0.17449 (18)	0.53435 (11)	0.0223 (7)	
H33	−0.0452	0.1523	0.5089	0.027*	
C34	0.1571 (3)	0.15222 (17)	0.54098 (10)	0.0188 (6)	
C35	0.2163 (3)	0.09796 (18)	0.51084 (11)	0.0221 (7)	
C035	0.1280 (3)	0.05593 (19)	0.46719 (11)	0.0233 (7)	
H3D	0.1859	0.0297	0.4484	0.035*	
H3E	0.0718	0.097	0.4481	0.035*	
H3F	0.0707	0.0147	0.4776	0.035*	
C36	0.3541 (3)	0.08390 (18)	0.52293 (10)	0.0215 (6)	
H36	0.3927	0.0499	0.5028	0.026*	
C037	0.5934 (3)	0.09776 (19)	0.57447 (11)	0.0246 (7)	
H3A	0.6361	0.1247	0.6037	0.037*	
H3B	0.6338	0.1176	0.5487	0.037*	
H3C	0.6063	0.0386	0.5777	0.037*	
C37	0.4428 (3)	0.11716 (17)	0.56384 (10)	0.0186 (6)	
C38	0.3866 (3)	0.16917 (16)	0.59365 (10)	0.0155 (6)	
C39	0.2449 (3)	0.18882 (17)	0.58077 (10)	0.0150 (6)	
C41	0.4751 (3)	0.33761 (17)	0.55930 (10)	0.0163 (6)	
H41	0.5404	0.2966	0.5694	0.02*	
C42	0.4644 (3)	0.37153 (18)	0.51340 (10)	0.0192 (6)	
H42	0.5205	0.3524	0.4933	0.023*	
C43	0.3705 (3)	0.43303 (17)	0.49844 (10)	0.0180 (6)	
H43	0.3619	0.4551	0.4678	0.022*	
C44	0.2867 (3)	0.46324 (17)	0.52910 (9)	0.0141 (6)	
C45	0.1898 (3)	0.52914 (17)	0.51813 (10)	0.0170 (6)	
C045	0.1656 (3)	0.57097 (19)	0.46935 (10)	0.0220 (7)	
H4D	0.086	0.6059	0.4657	0.033*	
H4E	0.1516	0.5292	0.4447	0.033*	
H4F	0.2437	0.6042	0.4668	0.033*	
C46	0.1203 (3)	0.55275 (17)	0.55295 (10)	0.0171 (6)	
H46	0.0571	0.5958	0.5459	0.021*	
C47	0.1387 (3)	0.51566 (16)	0.59901 (10)	0.0144 (6)	
C047	0.0584 (3)	0.54758 (17)	0.63478 (10)	0.0172 (6)	
H4A	0.0941	0.5236	0.6656	0.026*	
H4B	−0.036	0.5323	0.6247	0.026*	
H4C	0.0661	0.6071	0.6368	0.026*	
C48	0.2301 (3)	0.45009 (16)	0.60983 (9)	0.0126 (5)	
C49	0.3039 (3)	0.42446 (16)	0.57450 (9)	0.0120 (5)	
N1	0.4521 (3)	0.39864 (14)	0.73466 (8)	0.0163 (5)	
N2	0.5067 (2)	0.22294 (14)	0.72955 (8)	0.0132 (5)	
N3	0.2023 (2)	0.24606 (14)	0.61007 (8)	0.0149 (5)	
N4	0.3956 (2)	0.36196 (13)	0.58873 (8)	0.0134 (5)	
N5	0.7160 (3)	0.18009 (16)	0.41223 (10)	0.0304 (7)	
N6	0.2248 (3)	0.20101 (19)	0.39606 (12)	0.0384 (7)	
O1	0.58992 (19)	0.34870 (11)	0.67177 (6)	0.0138 (4)	
O2	0.2508 (2)	0.26785 (12)	0.70859 (7)	0.0160 (4)	
O3	0.4573 (2)	0.20388 (11)	0.63314 (7)	0.0150 (4)	
O4	0.25780 (19)	0.41113 (11)	0.65191 (6)	0.0142 (4)	
O5	0.6915 (3)	0.23919 (16)	0.48278 (10)	0.0424 (7)	
O6	0.0297 (3)	0.25457 (18)	0.41325 (11)	0.0560 (8)	
Hf1	0.388124 (12)	0.307882 (7)	0.666362 (4)	0.01319 (4)	

### Atomic displacement parameters (Å^2^)

**Table:**

	*U*^11^	*U*^22^	*U*^33^	*U*^12^	*U*^13^	*U*^23^
C01	0.041 (2)	0.030 (2)	0.061 (3)	0.0024 (18)	0.006 (2)	0.0053 (19)
C02	0.033 (2)	0.057 (3)	0.092 (4)	0.001 (2)	0.010 (2)	0.017 (3)
C03	0.054 (3)	0.058 (3)	0.041 (2)	0.000 (2)	0.013 (2)	0.004 (2)
C04	0.0207 (18)	0.0263 (18)	0.046 (2)	0.0006 (14)	−0.0064 (16)	−0.0087 (16)
C05	0.045 (2)	0.0272 (18)	0.036 (2)	0.0010 (16)	0.0037 (17)	−0.0005 (15)
C06	0.054 (3)	0.0322 (19)	0.036 (2)	−0.0063 (18)	0.0024 (18)	0.0033 (17)
C11	0.0246 (17)	0.0221 (15)	0.0157 (14)	0.0077 (13)	0.0060 (12)	0.0029 (12)
C12	0.046 (2)	0.0231 (16)	0.0135 (15)	0.0150 (15)	0.0085 (14)	0.0008 (12)
C13	0.049 (2)	0.0167 (15)	0.0127 (15)	0.0087 (15)	0.0019 (14)	−0.0014 (12)
C14	0.0348 (19)	0.0106 (13)	0.0125 (14)	0.0030 (12)	−0.0006 (13)	0.0012 (11)
C15	0.0360 (19)	0.0128 (14)	0.0184 (15)	−0.0035 (13)	−0.0038 (14)	0.0027 (12)
C015	0.047 (2)	0.0207 (16)	0.0216 (17)	−0.0062 (15)	−0.0025 (15)	−0.0028 (13)
C16	0.0280 (18)	0.0199 (15)	0.0223 (16)	−0.0042 (13)	−0.0039 (14)	0.0039 (13)
C017	0.0176 (16)	0.0263 (16)	0.0212 (16)	−0.0026 (13)	0.0015 (13)	0.0025 (13)
C17	0.0196 (16)	0.0140 (13)	0.0193 (15)	−0.0023 (11)	0.0007 (12)	0.0042 (11)
C18	0.0237 (16)	0.0103 (12)	0.0131 (14)	0.0024 (11)	0.0032 (12)	0.0036 (10)
C19	0.0261 (17)	0.0114 (13)	0.0136 (14)	0.0024 (12)	0.0020 (12)	0.0013 (11)
C21	0.0144 (14)	0.0158 (13)	0.0168 (14)	−0.0010 (12)	0.0051 (11)	0.0004 (11)
C22	0.0170 (15)	0.0171 (14)	0.0204 (15)	0.0042 (12)	0.0047 (12)	0.0029 (12)
C23	0.0242 (16)	0.0141 (13)	0.0130 (14)	0.0016 (12)	−0.0003 (12)	0.0026 (11)
C24	0.0198 (15)	0.0115 (12)	0.0119 (13)	−0.0010 (11)	0.0021 (11)	−0.0010 (10)
C25	0.0255 (17)	0.0136 (13)	0.0120 (13)	−0.0042 (12)	0.0051 (12)	0.0004 (11)
C025	0.0265 (17)	0.0204 (15)	0.0148 (14)	−0.0034 (12)	0.0051 (12)	0.0044 (12)
C26	0.0208 (16)	0.0235 (15)	0.0141 (14)	−0.0037 (12)	0.0071 (12)	0.0020 (12)
C027	0.0179 (17)	0.044 (2)	0.0255 (17)	0.0009 (14)	0.0103 (14)	0.0122 (15)
C27	0.0177 (15)	0.0212 (14)	0.0159 (14)	−0.0005 (12)	0.0050 (11)	0.0012 (12)
C28	0.0170 (15)	0.0144 (13)	0.0133 (13)	0.0001 (11)	0.0039 (11)	0.0003 (11)
C29	0.0156 (14)	0.0116 (12)	0.0099 (12)	0.0007 (11)	0.0039 (10)	−0.0013 (11)
C31	0.0188 (16)	0.0166 (14)	0.0174 (15)	0.0033 (12)	0.0048 (12)	0.0013 (11)
C32	0.0198 (17)	0.0188 (15)	0.0319 (18)	0.0025 (13)	0.0032 (14)	0.0037 (13)
C33	0.0220 (17)	0.0178 (15)	0.0243 (16)	−0.0017 (12)	−0.0019 (13)	−0.0015 (12)
C34	0.0261 (17)	0.0128 (13)	0.0171 (14)	−0.0012 (12)	0.0037 (12)	0.0021 (11)
C35	0.0294 (18)	0.0176 (15)	0.0193 (15)	−0.0021 (13)	0.0046 (13)	−0.0036 (12)
C035	0.0262 (18)	0.0232 (16)	0.0201 (15)	−0.0014 (13)	0.0040 (13)	−0.0075 (12)
C36	0.0322 (18)	0.0143 (14)	0.0195 (15)	0.0040 (13)	0.0089 (13)	−0.0044 (12)
C037	0.0282 (18)	0.0256 (16)	0.0211 (16)	0.0083 (14)	0.0075 (14)	−0.0043 (13)
C37	0.0234 (16)	0.0156 (14)	0.0180 (15)	0.0028 (12)	0.0071 (12)	0.0015 (11)
C38	0.0196 (15)	0.0117 (13)	0.0163 (14)	0.0014 (11)	0.0062 (12)	0.0043 (11)
C39	0.0197 (15)	0.0124 (12)	0.0140 (13)	−0.0012 (12)	0.0056 (11)	0.0021 (11)
C41	0.0170 (15)	0.0141 (13)	0.0182 (14)	0.0006 (11)	0.0049 (12)	−0.0004 (11)
C42	0.0234 (16)	0.0199 (14)	0.0170 (15)	−0.0025 (12)	0.0108 (12)	−0.0020 (12)
C43	0.0267 (17)	0.0170 (14)	0.0102 (13)	−0.0085 (12)	0.0036 (12)	0.0009 (11)
C44	0.0144 (14)	0.0141 (13)	0.0123 (13)	−0.0049 (11)	−0.0008 (11)	0.0001 (11)
C45	0.0172 (15)	0.0153 (13)	0.0155 (14)	−0.0063 (11)	−0.0040 (12)	0.0040 (11)
C045	0.0195 (16)	0.0205 (15)	0.0218 (15)	−0.0018 (12)	−0.0054 (12)	0.0095 (13)
C46	0.0123 (14)	0.0126 (13)	0.0234 (15)	0.0003 (11)	−0.0039 (12)	0.0053 (11)
C47	0.0127 (14)	0.0126 (13)	0.0170 (14)	−0.0012 (11)	0.0012 (11)	−0.0025 (11)
C047	0.0155 (15)	0.0158 (13)	0.0192 (15)	0.0029 (11)	0.0009 (12)	−0.0039 (11)
C48	0.0130 (14)	0.0117 (12)	0.0124 (13)	−0.0014 (10)	0.0009 (11)	−0.0002 (10)
C49	0.0126 (14)	0.0116 (12)	0.0115 (13)	−0.0029 (11)	0.0019 (10)	−0.0007 (10)
N1	0.0224 (14)	0.0144 (11)	0.0124 (12)	0.0053 (10)	0.0046 (10)	0.0034 (9)
N2	0.0175 (13)	0.0120 (10)	0.0108 (11)	0.0016 (9)	0.0043 (10)	0.0010 (9)
N3	0.0201 (13)	0.0114 (11)	0.0140 (12)	0.0015 (10)	0.0051 (10)	0.0028 (9)
N4	0.0156 (12)	0.0101 (10)	0.0151 (12)	0.0003 (9)	0.0048 (10)	0.0005 (9)
N5	0.0381 (17)	0.0220 (14)	0.0281 (15)	−0.0032 (12)	−0.0006 (13)	−0.0011 (12)
N6	0.0297 (17)	0.0367 (17)	0.049 (2)	0.0018 (14)	0.0087 (15)	0.0067 (15)
O1	0.0174 (11)	0.0137 (9)	0.0104 (9)	0.0009 (8)	0.0030 (8)	−0.0010 (7)
O2	0.0151 (10)	0.0195 (10)	0.0140 (10)	0.0038 (8)	0.0042 (8)	0.0054 (8)
O3	0.0185 (10)	0.0130 (9)	0.0134 (9)	0.0023 (8)	0.0029 (8)	0.0009 (8)
O4	0.0186 (10)	0.0147 (9)	0.0098 (9)	0.0050 (8)	0.0041 (8)	0.0018 (7)
O5	0.0346 (15)	0.0446 (16)	0.0445 (17)	0.0015 (12)	−0.0002 (13)	−0.0210 (13)
O6	0.0421 (18)	0.0466 (17)	0.079 (2)	0.0113 (14)	0.0119 (16)	0.0051 (16)
Hf1	0.01641 (6)	0.01255 (6)	0.01140 (6)	0.00269 (5)	0.00470 (4)	0.00157 (5)

### Geometric parameters (Å, °)

**Table:**

C01—O6	1.218 (5)	C027—H2D	0.96
C01—N6	1.337 (5)	C027—H2E	0.96
C01—H01	0.93	C027—H2F	0.96
C02—N6	1.450 (5)	C27—C28	1.389 (4)
C02—H02A	0.96	C28—O2	1.332 (3)
C02—H02B	0.96	C28—C29	1.417 (4)
C02—H02C	0.96	C29—N2	1.365 (3)
C03—N6	1.449 (5)	C31—N3	1.316 (4)
C03—H03A	0.96	C31—C32	1.409 (4)
C03—H03B	0.96	C31—H31	0.93
C03—H03C	0.96	C32—C33	1.362 (4)
C04—O5	1.226 (4)	C32—H32	0.93
C04—N5	1.330 (4)	C33—C34	1.413 (4)
C04—H04	0.93	C33—H33	0.93
C05—N5	1.442 (4)	C34—C39	1.414 (4)
C05—H05A	0.96	C34—C35	1.432 (4)
C05—H05B	0.96	C35—C36	1.367 (4)
C05—H05C	0.96	C35—C035	1.530 (4)
C06—N5	1.443 (5)	C035—H3D	0.96
C06—H06A	0.96	C035—H3E	0.96
C06—H06B	0.96	C035—H3F	0.96
C06—H06C	0.96	C36—C37	1.419 (4)
C11—N1	1.328 (4)	C36—H36	0.93
C11—C12	1.407 (4)	C037—C37	1.504 (4)
C11—H11	0.93	C037—H3A	0.96
C12—C13	1.368 (5)	C037—H3B	0.96
C12—H12	0.93	C037—H3C	0.96
C13—C14	1.414 (4)	C37—C38	1.387 (4)
C13—H13	0.93	C38—O3	1.325 (3)
C14—C15	1.417 (5)	C38—C39	1.423 (4)
C14—C19	1.426 (4)	C39—N3	1.366 (3)
C15—C16	1.381 (4)	C41—N4	1.324 (3)
C15—C015	1.518 (4)	C41—C42	1.401 (4)
C015—H1A	0.96	C41—H41	0.93
C015—H1B	0.96	C42—C43	1.369 (4)
C015—H1C	0.96	C42—H42	0.93
C16—C17	1.416 (4)	C43—C44	1.412 (4)
C16—H16	0.93	C43—H43	0.93
C017—C17	1.504 (4)	C44—C49	1.415 (4)
C017—H1D	0.96	C44—C45	1.425 (4)
C017—H1E	0.96	C45—C46	1.373 (4)
C017—H1F	0.96	C45—C045	1.519 (4)
C17—C18	1.375 (4)	C045—H4D	0.96
C18—O1	1.332 (3)	C045—H4E	0.96
C18—C19	1.417 (4)	C045—H4F	0.96
C19—N1	1.367 (4)	C46—C47	1.420 (4)
C21—N2	1.322 (4)	C46—H46	0.93
C21—C22	1.403 (4)	C47—C48	1.387 (4)
C21—H21	0.93	C47—C047	1.507 (4)
C22—C23	1.373 (4)	C047—H4A	0.96
C22—H22	0.93	C047—H4B	0.96
C23—C24	1.407 (4)	C047—H4C	0.96
C23—H23	0.93	C48—O4	1.332 (3)
C24—C29	1.416 (4)	C48—C49	1.423 (4)
C24—C25	1.428 (4)	C49—N4	1.364 (3)
C25—C26	1.367 (4)	N1—Hf1	2.413 (2)
C25—C025	1.515 (4)	N2—Hf1	2.377 (2)
C025—H2A	0.96	N3—Hf1	2.409 (2)
C025—H2B	0.96	N4—Hf1	2.393 (2)
C025—H2C	0.96	O1—Hf1	2.094 (2)
C26—C27	1.423 (4)	O2—Hf1	2.0981 (19)
C26—H26	0.93	O3—Hf1	2.1036 (19)
C027—C27	1.503 (4)	O4—Hf1	2.0964 (19)
			
O6—C01—N6	125.3 (4)	C33—C34—C35	126.0 (3)
O6—C01—H01	117.3	C39—C34—C35	118.1 (3)
N6—C01—H01	117.3	C36—C35—C34	117.7 (3)
N6—C02—H02A	109.5	C36—C35—C035	121.2 (3)
N6—C02—H02B	109.5	C34—C35—C035	121.1 (3)
H02A—C02—H02B	109.5	C35—C035—H3D	109.5
N6—C02—H02C	109.5	C35—C035—H3E	109.5
H02A—C02—H02C	109.5	H3D—C035—H3E	109.5
H02B—C02—H02C	109.5	C35—C035—H3F	109.5
N6—C03—H03A	109.5	H3D—C035—H3F	109.5
N6—C03—H03B	109.5	H3E—C035—H3F	109.5
H03A—C03—H03B	109.5	C35—C36—C37	125.1 (3)
N6—C03—H03C	109.5	C35—C36—H36	117.5
H03A—C03—H03C	109.5	C37—C36—H36	117.5
H03B—C03—H03C	109.5	C37—C037—H3A	109.5
O5—C04—N5	126.3 (3)	C37—C037—H3B	109.5
O5—C04—H04	116.9	H3A—C037—H3B	109.5
N5—C04—H04	116.9	C37—C037—H3C	109.5
N5—C05—H05A	109.5	H3A—C037—H3C	109.5
N5—C05—H05B	109.5	H3B—C037—H3C	109.5
H05A—C05—H05B	109.5	C38—C37—C36	117.9 (3)
N5—C05—H05C	109.5	C38—C37—C037	120.7 (3)
H05A—C05—H05C	109.5	C36—C37—C037	121.4 (3)
H05B—C05—H05C	109.5	O3—C38—C37	124.2 (3)
N5—C06—H06A	109.5	O3—C38—C39	117.2 (2)
N5—C06—H06B	109.5	C37—C38—C39	118.6 (3)
H06A—C06—H06B	109.5	N3—C39—C34	123.5 (3)
N5—C06—H06C	109.5	N3—C39—C38	114.0 (2)
H06A—C06—H06C	109.5	C34—C39—C38	122.5 (3)
H06B—C06—H06C	109.5	N4—C41—C42	122.2 (3)
N1—C11—C12	121.8 (3)	N4—C41—H41	118.9
N1—C11—H11	119.1	C42—C41—H41	118.9
C12—C11—H11	119.1	C43—C42—C41	119.4 (3)
C13—C12—C11	120.4 (3)	C43—C42—H42	120.3
C13—C12—H12	119.8	C41—C42—H42	120.3
C11—C12—H12	119.8	C42—C43—C44	120.6 (3)
C12—C13—C14	120.0 (3)	C42—C43—H43	119.7
C12—C13—H13	120	C44—C43—H43	119.7
C14—C13—H13	120	C43—C44—C49	115.9 (3)
C13—C14—C15	125.9 (3)	C43—C44—C45	125.4 (3)
C13—C14—C19	115.8 (3)	C49—C44—C45	118.7 (3)
C15—C14—C19	118.3 (3)	C46—C45—C44	117.7 (3)
C16—C15—C14	118.0 (3)	C46—C45—C045	121.9 (3)
C16—C15—C015	121.0 (3)	C44—C45—C045	120.4 (3)
C14—C15—C015	120.9 (3)	C45—C045—H4D	109.5
C15—C015—H1A	109.5	C45—C045—H4E	109.5
C15—C015—H1B	109.5	H4D—C045—H4E	109.5
H1A—C015—H1B	109.5	C45—C045—H4F	109.5
C15—C015—H1C	109.5	H4D—C045—H4F	109.5
H1A—C015—H1C	109.5	H4E—C045—H4F	109.5
H1B—C015—H1C	109.5	C45—C46—C47	124.2 (3)
C15—C16—C17	124.1 (3)	C45—C46—H46	117.9
C15—C16—H16	118	C47—C46—H46	117.9
C17—C16—H16	118	C48—C47—C46	118.8 (3)
C17—C017—H1D	109.5	C48—C47—C047	121.5 (2)
C17—C017—H1E	109.5	C46—C47—C047	119.7 (2)
H1D—C017—H1E	109.5	C47—C047—H4A	109.5
C17—C017—H1F	109.5	C47—C047—H4B	109.5
H1D—C017—H1F	109.5	H4A—C047—H4B	109.5
H1E—C017—H1F	109.5	C47—C047—H4C	109.5
C18—C17—C16	118.5 (3)	H4A—C047—H4C	109.5
C18—C17—C017	119.4 (3)	H4B—C047—H4C	109.5
C16—C17—C017	122.1 (3)	O4—C48—C47	124.4 (2)
O1—C18—C17	123.8 (3)	O4—C48—C49	117.3 (2)
O1—C18—C19	117.1 (3)	C47—C48—C49	118.2 (2)
C17—C18—C19	119.2 (3)	N4—C49—C44	123.2 (2)
N1—C19—C18	114.7 (2)	N4—C49—C48	114.5 (2)
N1—C19—C14	123.5 (3)	C44—C49—C48	122.3 (2)
C18—C19—C14	121.8 (3)	C11—N1—C19	118.4 (3)
N2—C21—C22	122.5 (3)	C11—N1—Hf1	128.4 (2)
N2—C21—H21	118.7	C19—N1—Hf1	113.17 (17)
C22—C21—H21	118.7	C21—N2—C29	118.5 (2)
C23—C22—C21	119.3 (3)	C21—N2—Hf1	127.59 (18)
C23—C22—H22	120.3	C29—N2—Hf1	113.74 (17)
C21—C22—H22	120.3	C31—N3—C39	118.2 (2)
C22—C23—C24	120.2 (3)	C31—N3—Hf1	128.55 (19)
C22—C23—H23	119.9	C39—N3—Hf1	113.09 (18)
C24—C23—H23	119.9	C41—N4—C49	118.7 (2)
C23—C24—C29	116.3 (2)	C41—N4—Hf1	127.79 (19)
C23—C24—C25	125.2 (3)	C49—N4—Hf1	113.50 (17)
C29—C24—C25	118.4 (3)	C04—N5—C05	120.5 (3)
C26—C25—C24	117.5 (3)	C04—N5—C06	122.7 (3)
C26—C25—C025	121.8 (3)	C05—N5—C06	116.8 (3)
C24—C25—C025	120.7 (3)	C01—N6—C03	121.1 (3)
C25—C025—H2A	109.5	C01—N6—C02	122.2 (4)
C25—C025—H2B	109.5	C03—N6—C02	116.7 (4)
H2A—C025—H2B	109.5	C18—O1—Hf1	124.59 (17)
C25—C025—H2C	109.5	C28—O2—Hf1	123.09 (17)
H2A—C025—H2C	109.5	C38—O3—Hf1	123.35 (17)
H2B—C025—H2C	109.5	C48—O4—Hf1	123.83 (16)
C25—C26—C27	124.9 (3)	O1—Hf1—O4	108.44 (8)
C25—C26—H26	117.5	O1—Hf1—O2	141.52 (7)
C27—C26—H26	117.5	O4—Hf1—O2	84.51 (8)
C27—C027—H2D	109.5	O1—Hf1—O3	83.35 (8)
C27—C027—H2E	109.5	O4—Hf1—O3	141.82 (7)
H2D—C027—H2E	109.5	O2—Hf1—O3	109.03 (8)
C27—C027—H2F	109.5	O1—Hf1—N2	78.40 (8)
H2D—C027—H2F	109.5	O4—Hf1—N2	142.90 (7)
H2E—C027—H2F	109.5	O2—Hf1—N2	70.87 (8)
C28—C27—C26	118.0 (3)	O3—Hf1—N2	74.17 (7)
C28—C27—C027	120.8 (3)	O1—Hf1—N4	75.30 (7)
C26—C27—C027	121.1 (3)	O4—Hf1—N4	70.39 (7)
O2—C28—C27	123.5 (3)	O2—Hf1—N4	141.93 (8)
O2—C28—C29	117.9 (2)	O3—Hf1—N4	78.33 (7)
C27—C28—C29	118.5 (3)	N2—Hf1—N4	143.72 (8)
N2—C29—C24	123.0 (2)	O1—Hf1—N3	141.10 (7)
N2—C29—C28	114.3 (2)	O4—Hf1—N3	80.26 (8)
C24—C29—C28	122.6 (2)	O2—Hf1—N3	75.64 (8)
N3—C31—C32	122.5 (3)	O3—Hf1—N3	69.58 (8)
N3—C31—H31	118.7	N2—Hf1—N3	117.89 (8)
C32—C31—H31	118.7	N4—Hf1—N3	72.32 (8)
C33—C32—C31	119.5 (3)	O1—Hf1—N1	69.92 (8)
C33—C32—H32	120.2	O4—Hf1—N1	74.58 (8)
C31—C32—H32	120.2	O2—Hf1—N1	79.61 (8)
C32—C33—C34	120.2 (3)	O3—Hf1—N1	141.74 (8)
C32—C33—H33	119.9	N2—Hf1—N1	74.00 (8)
C34—C33—H33	119.9	N4—Hf1—N1	118.34 (8)
C33—C34—C39	115.9 (3)	N3—Hf1—N1	146.09 (8)
			
N1—C11—C12—C13	−1.5 (4)	C32—C31—N3—C39	−0.9 (4)
C11—C12—C13—C14	−0.8 (4)	C32—C31—N3—Hf1	173.9 (2)
C12—C13—C14—C15	−177.4 (3)	C34—C39—N3—C31	4.0 (4)
C12—C13—C14—C19	3.2 (4)	C38—C39—N3—C31	−176.2 (2)
C13—C14—C15—C16	−178.6 (3)	C34—C39—N3—Hf1	−171.6 (2)
C19—C14—C15—C16	0.7 (4)	C38—C39—N3—Hf1	8.2 (3)
C13—C14—C15—C015	1.6 (4)	C42—C41—N4—C49	−2.6 (4)
C19—C14—C15—C015	−179.0 (3)	C42—C41—N4—Hf1	176.4 (2)
C14—C15—C16—C17	1.9 (4)	C44—C49—N4—C41	1.9 (4)
C015—C15—C16—C17	−178.4 (3)	C48—C49—N4—C41	−175.8 (2)
C15—C16—C17—C18	−1.5 (4)	C44—C49—N4—Hf1	−177.3 (2)
C15—C16—C17—C017	177.6 (3)	C48—C49—N4—Hf1	5.0 (3)
C16—C17—C18—O1	177.7 (2)	O5—C04—N5—C05	−3.6 (6)
C017—C17—C18—O1	−1.4 (4)	O5—C04—N5—C06	176.6 (3)
C16—C17—C18—C19	−1.6 (4)	O6—C01—N6—C03	0.0 (6)
C017—C17—C18—C19	179.4 (3)	O6—C01—N6—C02	−179.7 (4)
O1—C18—C19—N1	4.2 (4)	C17—C18—O1—Hf1	171.5 (2)
C17—C18—C19—N1	−176.5 (2)	C19—C18—O1—Hf1	−9.2 (3)
O1—C18—C19—C14	−175.1 (2)	C27—C28—O2—Hf1	−179.5 (2)
C17—C18—C19—C14	4.2 (4)	C29—C28—O2—Hf1	−1.8 (3)
C13—C14—C19—N1	−3.6 (4)	C37—C38—O3—Hf1	161.2 (2)
C15—C14—C19—N1	177.0 (3)	C39—C38—O3—Hf1	−17.1 (3)
C13—C14—C19—C18	175.7 (3)	C47—C48—O4—Hf1	176.5 (2)
C15—C14—C19—C18	−3.8 (4)	C49—C48—O4—Hf1	−6.2 (3)
N2—C21—C22—C23	−1.5 (4)	C18—O1—Hf1—O4	72.3 (2)
C21—C22—C23—C24	0.7 (4)	C18—O1—Hf1—O2	−32.6 (2)
C22—C23—C24—C29	1.1 (4)	C18—O1—Hf1—O3	−145.0 (2)
C22—C23—C24—C25	178.7 (3)	C18—O1—Hf1—N2	−69.82 (19)
C23—C24—C25—C26	−175.7 (3)	C18—O1—Hf1—N4	135.4 (2)
C29—C24—C25—C26	1.9 (4)	C18—O1—Hf1—N3	169.79 (17)
C23—C24—C25—C025	3.6 (4)	C18—O1—Hf1—N1	7.20 (18)
C29—C24—C25—C025	−178.9 (2)	C48—O4—Hf1—O1	72.7 (2)
C24—C25—C26—C27	0.3 (4)	C48—O4—Hf1—O2	−144.5 (2)
C025—C25—C26—C27	−178.9 (3)	C48—O4—Hf1—O3	−30.4 (3)
C25—C26—C27—C28	−1.7 (4)	C48—O4—Hf1—N2	167.82 (18)
C25—C26—C27—C027	176.1 (3)	C48—O4—Hf1—N4	6.44 (19)
C26—C27—C28—O2	178.6 (3)	C48—O4—Hf1—N3	−68.1 (2)
C027—C27—C28—O2	0.7 (4)	C48—O4—Hf1—N1	134.8 (2)
C26—C27—C28—C29	0.9 (4)	C28—O2—Hf1—O1	−37.5 (3)
C027—C27—C28—C29	−176.9 (3)	C28—O2—Hf1—O4	−150.5 (2)
C23—C24—C29—N2	−2.2 (4)	C28—O2—Hf1—O3	66.2 (2)
C25—C24—C29—N2	−180.0 (2)	C28—O2—Hf1—N2	1.34 (19)
C23—C24—C29—C28	175.0 (3)	C28—O2—Hf1—N4	161.55 (18)
C25—C24—C29—C28	−2.7 (4)	C28—O2—Hf1—N3	128.2 (2)
O2—C28—C29—N2	1.0 (4)	C28—O2—Hf1—N1	−75.2 (2)
C27—C28—C29—N2	178.8 (2)	C38—O3—Hf1—O1	−136.1 (2)
O2—C28—C29—C24	−176.5 (2)	C38—O3—Hf1—O4	−24.6 (2)
C27—C28—C29—C24	1.3 (4)	C38—O3—Hf1—O2	81.4 (2)
N3—C31—C32—C33	−2.4 (4)	C38—O3—Hf1—N2	144.1 (2)
C31—C32—C33—C34	2.6 (4)	C38—O3—Hf1—N4	−59.77 (19)
C32—C33—C34—C39	0.3 (4)	C38—O3—Hf1—N3	15.51 (19)
C32—C33—C34—C35	−178.5 (3)	C38—O3—Hf1—N1	178.84 (18)
C33—C34—C35—C36	179.8 (3)	C21—N2—Hf1—O1	−28.5 (2)
C39—C34—C35—C36	1.0 (4)	C29—N2—Hf1—O1	155.80 (19)
C33—C34—C35—C035	−1.4 (5)	C21—N2—Hf1—O4	−133.8 (2)
C39—C34—C35—C035	179.9 (3)	C29—N2—Hf1—O4	50.5 (2)
C34—C35—C36—C37	1.8 (5)	C21—N2—Hf1—O2	175.0 (2)
C035—C35—C36—C37	−177.1 (3)	C29—N2—Hf1—O2	−0.71 (17)
C35—C36—C37—C38	−1.1 (4)	C21—N2—Hf1—O3	57.8 (2)
C35—C36—C37—C037	179.6 (3)	C29—N2—Hf1—O3	−117.90 (19)
C36—C37—C38—O3	179.4 (2)	C21—N2—Hf1—N4	15.6 (3)
C037—C37—C38—O3	−1.3 (4)	C29—N2—Hf1—N4	−160.04 (16)
C36—C37—C38—C39	−2.3 (4)	C21—N2—Hf1—N3	113.7 (2)
C037—C37—C38—C39	177.0 (3)	C29—N2—Hf1—N3	−61.99 (19)
C33—C34—C39—N3	−3.7 (4)	C21—N2—Hf1—N1	−100.7 (2)
C35—C34—C39—N3	175.2 (3)	C29—N2—Hf1—N1	83.61 (18)
C33—C34—C39—C38	176.6 (3)	C41—N4—Hf1—O1	58.9 (2)
C35—C34—C39—C38	−4.5 (4)	C49—N4—Hf1—O1	−122.02 (19)
O3—C38—C39—N3	3.8 (3)	C41—N4—Hf1—O4	175.0 (2)
C37—C38—C39—N3	−174.5 (2)	C49—N4—Hf1—O4	−5.88 (17)
O3—C38—C39—C34	−176.4 (2)	C41—N4—Hf1—O2	−133.3 (2)
C37—C38—C39—C34	5.2 (4)	C49—N4—Hf1—O2	45.8 (2)
N4—C41—C42—C43	1.2 (4)	C41—N4—Hf1—O3	−27.2 (2)
C41—C42—C43—C44	1.1 (4)	C49—N4—Hf1—O3	151.88 (19)
C42—C43—C44—C49	−1.8 (4)	C41—N4—Hf1—N2	14.0 (3)
C42—C43—C44—C45	177.3 (3)	C49—N4—Hf1—N2	−166.89 (16)
C43—C44—C45—C46	−177.6 (3)	C41—N4—Hf1—N3	−99.3 (2)
C49—C44—C45—C46	1.4 (4)	C49—N4—Hf1—N3	79.83 (19)
C43—C44—C45—C045	2.5 (4)	C41—N4—Hf1—N1	115.8 (2)
C49—C44—C45—C045	−178.5 (2)	C49—N4—Hf1—N1	−65.0 (2)
C44—C45—C46—C47	−0.1 (4)	C31—N3—Hf1—O1	−138.2 (2)
C045—C45—C46—C47	179.8 (3)	C39—N3—Hf1—O1	36.8 (2)
C45—C46—C47—C48	−1.5 (4)	C31—N3—Hf1—O4	−30.8 (2)
C45—C46—C47—C047	179.0 (3)	C39—N3—Hf1—O4	144.20 (18)
C46—C47—C48—O4	178.9 (2)	C31—N3—Hf1—O2	55.9 (2)
C047—C47—C48—O4	−1.6 (4)	C39—N3—Hf1—O2	−129.03 (18)
C46—C47—C48—C49	1.6 (4)	C31—N3—Hf1—O3	173.0 (2)
C047—C47—C48—C49	−178.9 (2)	C39—N3—Hf1—O3	−12.00 (17)
C43—C44—C49—N4	0.3 (4)	C31—N3—Hf1—N2	114.7 (2)
C45—C44—C49—N4	−178.8 (2)	C39—N3—Hf1—N2	−70.24 (19)
C43—C44—C49—C48	177.8 (2)	C31—N3—Hf1—N4	−103.2 (2)
C45—C44—C49—C48	−1.3 (4)	C39—N3—Hf1—N4	71.81 (18)
O4—C48—C49—N4	−0.1 (4)	C31—N3—Hf1—N1	11.5 (3)
C47—C48—C49—N4	177.4 (2)	C39—N3—Hf1—N1	−173.43 (16)
O4—C48—C49—C44	−177.8 (2)	C11—N1—Hf1—O1	175.0 (2)
C47—C48—C49—C44	−0.2 (4)	C19—N1—Hf1—O1	−4.19 (17)
C12—C11—N1—C19	1.2 (4)	C11—N1—Hf1—O4	58.2 (2)
C12—C11—N1—Hf1	−178.0 (2)	C19—N1—Hf1—O4	−120.94 (19)
C18—C19—N1—C11	−177.9 (2)	C11—N1—Hf1—O2	−28.9 (2)
C14—C19—N1—C11	1.5 (4)	C19—N1—Hf1—O2	151.94 (19)
C18—C19—N1—Hf1	1.4 (3)	C11—N1—Hf1—O3	−136.5 (2)
C14—C19—N1—Hf1	−179.3 (2)	C19—N1—Hf1—O3	44.3 (2)
C22—C21—N2—C29	0.4 (4)	C11—N1—Hf1—N2	−101.8 (2)
C22—C21—N2—Hf1	−175.1 (2)	C19—N1—Hf1—N2	79.04 (18)
C24—C29—N2—C21	1.5 (4)	C11—N1—Hf1—N4	115.2 (2)
C28—C29—N2—C21	−176.0 (2)	C19—N1—Hf1—N4	−63.9 (2)
C24—C29—N2—Hf1	177.6 (2)	C11—N1—Hf1—N3	14.6 (3)
C28—C29—N2—Hf1	0.1 (3)	C19—N1—Hf1—N3	−164.50 (16)

### Hydrogen-bond geometry (Å, °)

**Table:**

*D*—H···*A*	*D*—H	H···*A*	*D*···*A*	*D*—H···*A*
C42—H42···O5	0.93	2.55	3.348 (4)	144.
